# The Significance of Hydrogen Sulfide for Arabidopsis Seed Germination

**DOI:** 10.3389/fpls.2016.00930

**Published:** 2016-06-27

**Authors:** Emmanuel Baudouin, Aurélie Poilevey, Nishodi Indiketi Hewage, Françoise Cochet, Juliette Puyaubert, Christophe Bailly

**Affiliations:** Sorbonne Universités, Université Pierre et Marie Curie (UPMC), CNRS, Institut de Biologie Paris-Seine (IBPS), Unité de Biologie du DéveloppementParis, France

**Keywords:** hydrogen sulfide, seed germination, seed dormancy, Arabidopsis, cysteine desulfhydrase

## Abstract

Hydrogen sulfide (H_2_S) recently emerged as an important gaseous signaling molecule in plants. In this study, we investigated the possible functions of H_2_S in regulating Arabidopsis seed germination. NaHS treatments delayed seed germination in a dose-dependent manner and were ineffective in releasing seed dormancy. Interestingly, endogenous H_2_S content was enhanced in germinating seeds. This increase was correlated with higher activity of three enzymes (L-cysteine desulfhydrase, D-cysteine desulfhydrase, and β-cyanoalanine synthase) known as sources of H_2_S in plants. The H_2_S scavenger hypotaurine and the D/L cysteine desulfhydrase inhibitor propargylglycine significantly delayed seed germination. We analyzed the germinative capacity of *des1* seeds mutated in Arabidopsis cytosolic L-cysteine desulfhydrase. Although the mutant seeds do not exhibit germination-evoked H_2_S formation, they retained similar germination capacity as the wild-type seeds. In addition, *des1* seeds responded similarly to temperature and were as sensitive to ABA as wild type seeds. Taken together, these data suggest that, although its metabolism is stimulated upon seed imbibition, H_2_S plays, if any, a marginal role in regulating Arabidopsis seed germination under standard conditions.

## Introduction

Hydrogen sulfide (H_2_S) is a toxic gas found in volcanic emissions or produced in waterlogged wetlands. It is also formed during human activities including agriculture and car use. Plants are therefore subjected to H_2_S exposure during their whole lifespan. Although acute exposure to H_2_S has long been known as deleterious for plants (Lisjak et al., 2013), numerous reports also indicate that low doses of H_2_S might be beneficial for plant fitness. For instance, exogenous treatments with H_2_S-releasing chemicals such as sodium hydrosulfide (NaHS) improve the tolerance to abiotic stresses such as drought, salinity, heavy metals, or high temperature in a variety of plant species (Jin et al., 2011; Christou et al., 2013, 2014; Shen et al., 2013; Shi et al., 2013). The mechanisms for H_2_S alleviation of abiotic stress effects unraveled so far are essentially the stimulation of antioxidant defense. For instance, the activities of superoxide dismutase, catalase, and ascorbate peroxidase are enhanced by NaHS treatments and contribute to maintain low levels of H_2_O_2_ (for review, Li, 2013). In addition, wheat plants water-stressed in the presence of NaHS display higher ascorbate and glutathione contents, as well as a higher proportion of reduced forms (Shan et al., 2011). Besides stimulating antioxidant defense, exogenous H_2_S treatments also trigger stomata movements which might participate in plant adaptive response to water and osmotic stress (García-Mata and Lamattina, 2010; Jin et al., 2013; Honda et al., 2015). Nevertheless, as opposite effects were observed i.e., the promotion of either stomata closure or opening (García-Mata and Lamattina, 2010; Lisjak et al., 2010), the outcome of such exogenous treatments might be conditioned by the overall plant physiological status.

Recent data raised further interest toward H_2_S in plants by highlighting a signaling function of endogenously produced H_2_S (for review, Lisjak et al., 2013). Three enzymes have been identified as sources of H_2_S in plants (Riemenschneider et al., 2005b; Alvarez et al., 2010; García et al., 2010). L- and D-cysteine desulfhydrases (L/D-CDes) produce H_2_S as a by-product of L/D-cysteine degradation. On the other hand, β-cyanoalanine synthase (β-CAS) catalyzes the conversion of cysteine and cyanide to β-cyanoalanine and H_2_S. β-CAS activity is located in the mitochondria when desulfhydrases have been reported in mitochondria and cytosol (Romero et al., 2014). Of particular interest, Alvarez et al. (2010) characterized a cytosolic L-CDes designated DES1 that modulates the generation of H_2_S in the cytosol. The corresponding *des1* mutants exhibit a reduced H_2_S content that is correlated with a premature leaf senescence and the overexpression of senescence associated genes (Álvarez et al., 2012a). Further, investigations also indicated that *des1* mutants are more sensitive to drought, in good accordance with their impaired ABA-responsive stomatal closure (Jin et al., 2013). More globally, *des1* mutants appear more sensitive to abiotic and biotic stresses and present a lower induction of stress-responsive genes (Shi et al., 2015). Altogether it is now obvious that data obtained by manipulating exogenous or endogenous H_2_S level converge and plead for an important role of this compound in the regulation of plant response under stress.

Whereas the involvement of H_2_S in plants under stress is well established, its function in plant development has been far less investigated. In that sense, seed germination represents an interesting study case. Indeed germination being the first step of plant life cycle is crucial for the establishment of a robust plantlet. Under natural conditions, it also determines the efficiency of wild species to proliferate in ecosystems. The ability to germinate is therefore tightly controlled to occur only when environmental conditions get favorable for the successful development of the plantlet. In most species, mature seeds are dormant i.e., are unable to germinate under favorable conditions, and germination capacity is acquired only after seed dormancy has been released. Seed dormancy release and germination are therefore the two integral faces of a same coin when considering freshly harvested seeds. Dormancy release is controlled by a complex set of environmental, e.g., temperature or light, and internal, e.g., hormones, signals (Shu et al., 2016). When successful, germination is completed within a short window that starts with seed imbibition and ends up with radicle protrusion.

Several studies have addressed the effect of H_2_S treatment on seed germination (Zhang et al., 2008, 2010a,b; Li et al., 2012; Dooley et al., 2013). Nevertheless, in most of the cases, only combined effects of H_2_S and stress (e.g., osmotic, oxidative, or metal stress) have been reported. When studied alone (Zhang et al., 2010a; Dooley et al., 2013), contrasting effects of H_2_S were observed. Zhang et al. (2010a) observed no differences of wheat germination in the absence or presence of up to 1.5 mM NaHS. On the other hand, Dooley et al. (2013) reported a stimulation of germination in the four species analyzed (i.e., wheat, corn, pea, and bean). Noteworthy, in most of the cases (species, NaHS concentration), NaHS treatment only accelerated germination but hardly modify final germination efficiency (Dooley et al., 2013). Although these data suggest that modifications of H_2_S availability during seed imbibition could affect seed germination, no information on a possible role for endogenously evoked H_2_S have been reported. In the present paper, we further investigated the metabolism and possible involvement of H_2_S in Arabidopsis seeds germinated under standard conditions.

## Materials and methods

### Plant material and germination assays

Arabidopsis (*Arabidopsis thaliana*) seeds of Columbia-0 (Col-0) ecotype were used throughout the study. The *des1-1* mutant (SALK_103855) presents a T-DNA insertion in the second exon of *DES1* gene (At5g28030*)* leading to a knockout mutation (Alvarez et al., 2010). Col-0 and *des1-1* seeds were propagated simultaneously as previously described (Basbouss-Serhal et al., 2015). Plants were grown in a climate chamber (20/22°C under long day photoperiod (16 h light/8 h dark)) on a soil/perlite (2:1) mixture. Floral stems were cut at silique maturity (about 3 months after sowing) and dried for 4 days at room temperature in paper envelopes before seeds were collected. Freshly harvested seeds were stored at –20°C to preserve their dormancy.

Seed germination was tested in 9-cm Petri dishes (50 seeds per dish, three replicates) on a filter paper on the top of a layer of cotton wool moistened with 20 mL of deionized water or indicated solutions. Plated seeds were subsequently kept in the dark in growth chambers at the required temperature. Germination was scored over time, a seed being considered as germinated when the radicle protruded through the testa. All the chemicals assayed during germination tests were directly dissolved in the deionized water used for seed imbibition.

### Chemicals

D-cysteine (C8005), hypotaurine (H1384), and DL-propargylglycine (P7888) were purchased from Sigma-Aldrich (L'Isle d'Abeau Chesnes, France).

### Enzyme activity measurements

Seed material (30 mg of dry seeds per condition before imbibition) consisting of dry seeds or seeds imbibed for 6, 16, or 24 h was collected by rapid freezing in liquid nitrogen. After grinding in liquid nitrogen, soluble proteins were extracted from seed powder by the addition of 1 mL of 50 mM Tris-HCl (pH 8) containing 2.5 mM DTT and 10 μL of protease inhibitor cocktail (P9599, Sigma-Aldrich, L'Isle d'Abeau Chesnes, France). Following centrifugation (14,000 g, 4°C, 45 min), protein content was determined and was adjusted to 1 mg.mL^−1^.

L-cysteine desulfhydrase (L-CDes) activity was assayed essentially as previously described (Riemenschneider et al., 2005a). Reactions were performed in 1 mL final volume of 0.1 M Tris-HCl (pH 9), 2.5 mM DTT, 0.8 mM L-cysteine, and 50 μg of soluble proteins. Following incubation at 37°C for 15 min, reactions were stopped by the addition of 100 μL of 20 mM *N,N*-dimethyl-*p*-phenylenediamine (in 7.2 N HCl) and 100 μL of 30 mM FeCl_3_ (in 1.2 N HCl). After 10 min incubation at room temperature and centrifugation (14,000 g, 5 min), the formation of methylene blue was quantified at 670 nm. For the calculation of H_2_S formation, known quantities of NaHS were assayed in the same conditions, and used for standard curve determination.

D-cysteine desulfhydrase (D-CDes) was assayed in the conditions described for L-CDes activity except that Tris-HCl buffer was pH 8 and L-cysteine was replaced by D-Cysteine.

β-cyanoalanine synthase (β-CAS) activity was assayed as described in Meyer et al. (2003). Reactions were performed in 1 mL final volume of 0.1 M Tris-HCl (pH 9), 0.8 mM L-cysteine, 10 mM KCN and 100 μg of soluble proteins. Following incubation at 30°C for 15 min, reactions were stopped and subsequently processed as described above for L-CDes activity.

### Hydrogen sulfide quantification

Seed endogenous hydrogen quantification was adapted from Christou et al. (2013). Dry or imbibed seeds (30 mg) were ground in liquid nitrogen and seed powder was resuspended in 500 μL of 100 mM potassium phosphate buffer (pH 7) containing 10 mM EDTA. Following centrifugation (14,000 g, 4°C, 15 min), H_2_S content from 100 μL supernatant was measured in a final volume of 2 mL containing 100 mM potassium phosphate buffer (pH 7), 10 mM EDTA, 0.2 mM 5,5′-dithiobis(2-nitrobenzoic acid). After 5 min incubation at room temperature, the absorbance was determined at 412 nm. H_2_S quantity was deduced from a standard curve obtained with known NaHS concentrations.

## Results

### Exogenous NaHS treatments delay arabidopsis seed germination

**Figure 1:**
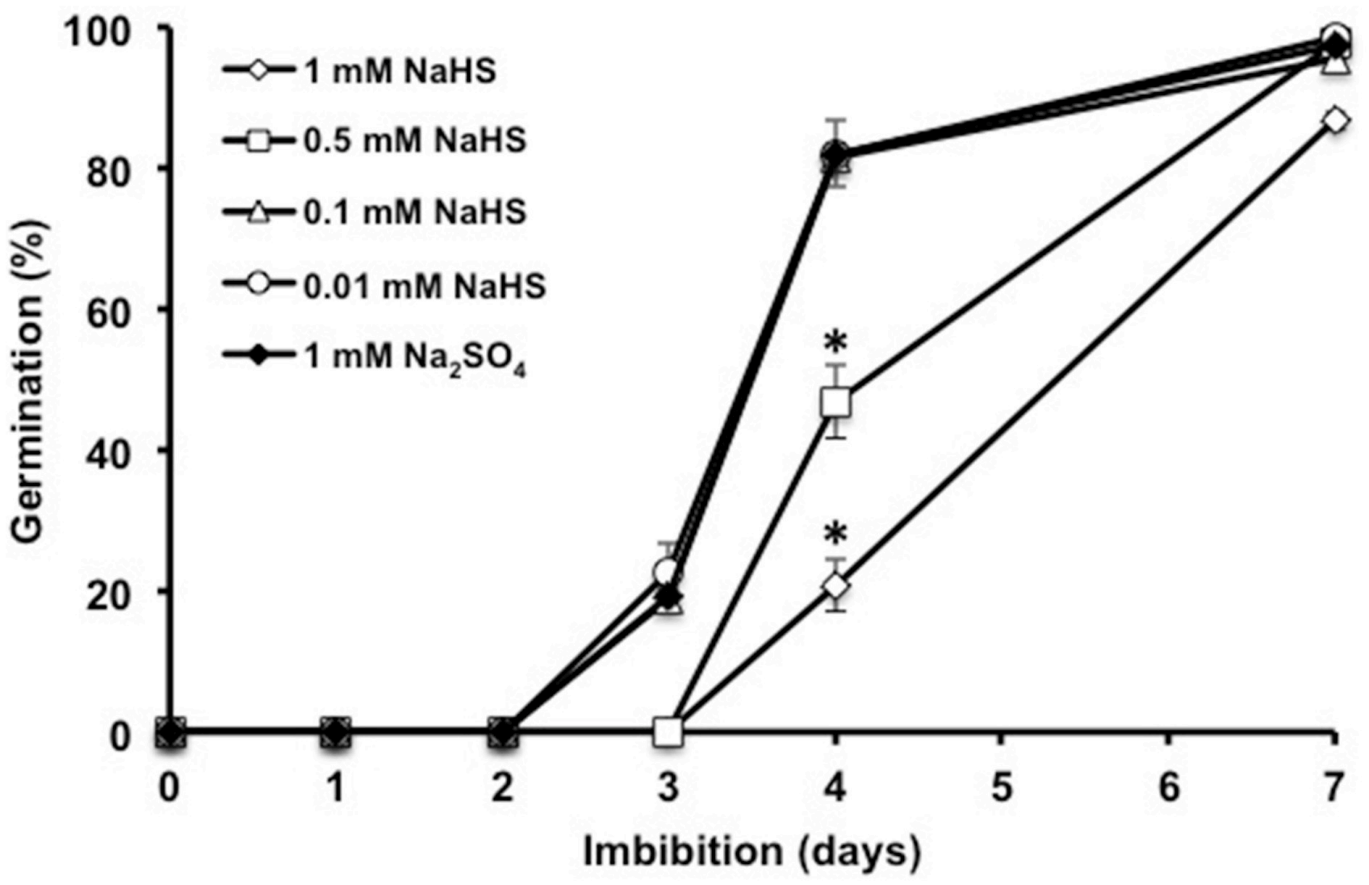
**Effect of the H_**2**_S donor NaHS on Arabidopsis seed germination**. Seeds (50 per condition) were imbibed on paper filters soaked with distilled water containing 1 mM Na_2_SO_4_ (control, close diamonds), 0.01 mM (open circles), 0.1 mM (open triangles), 0.5 mM (open squares), or 1 mM NaHS (open diamonds). Germination was recorded after incubation at 15°C in the dark for the indicated durations. Values are the mean ± *S.E*. of six experiments. Asterisks represent statistical differences relative to control at the same time point (Student's test; *P < 0.05).

Recent data suggested that treating seeds with H_2_S-releasing chemicals such as sodium hydrosulfide (NaHS) might stimulate seed germination in different legume and cereal species (Dooley et al., 2013). Using freshly harvested Arabidopsis seeds, germination was compared in the absence or presence of various concentrations of NaHS. As shown on Figure 1, germination was not affected when up to 100 μM NaHS was applied, but was delayed by higher concentrations, in a dose-dependent manner. When imbibed at 25°C, seeds did not germinate whatever the concentration of NaHS was, indicating that seed dormancy cannot be alleviated by NaHS treatment (data not shown). These data suggest that exogenously applied H_2_S, when efficient, negatively impacts Arabidopsis germination.

### Endogenous H_2_S level increases during seed imbibition

**Figure 2:**
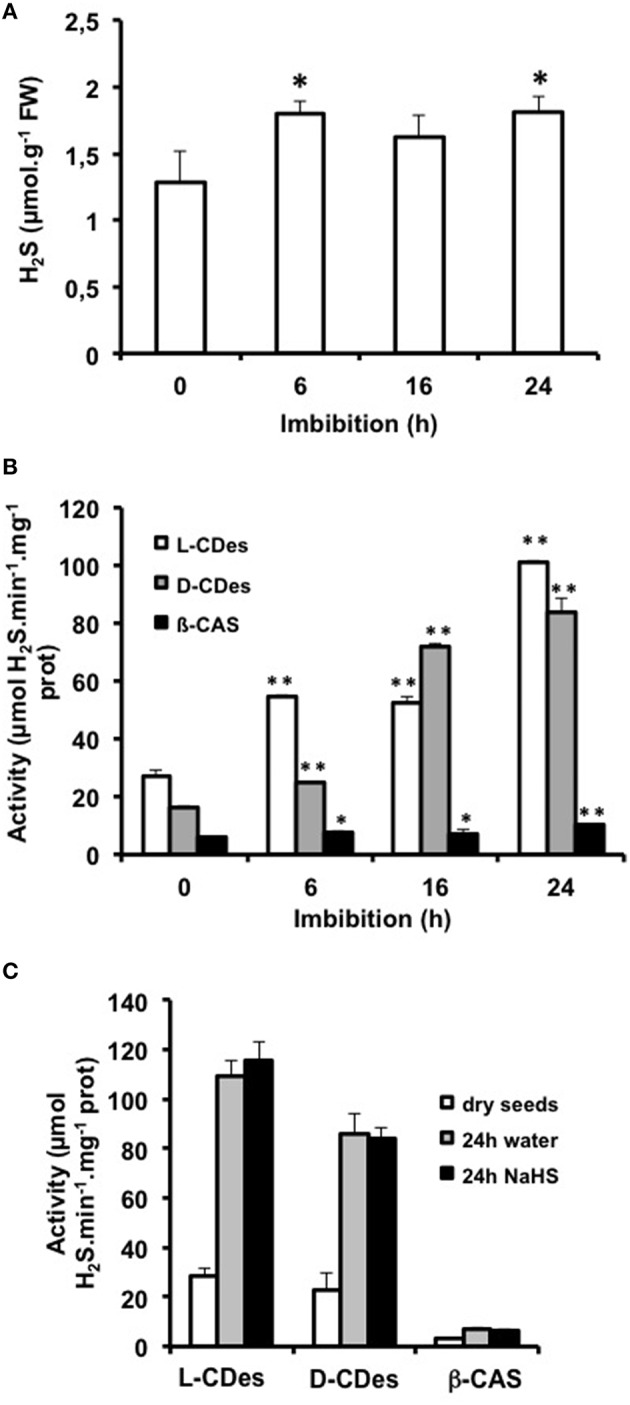
**H_**2**_S synthesis during Arabidopsis seed imbibition. (A)** Evolution of Arabidopsis seed H_2_S content during imbibition. Seeds were imbibed on distilled water in the dark at 15°C. Values are mean ± *S.E*. of three independent experiments. Asterisks represent statistical differences relative to H_2_S content in dry seeds (Student's test; *P < 0.05); **(B)** Evolution of the activities of H_2_S-generating enzymes in imbibed Arabidopsis seeds. Seeds were imbibed on distilled water in the dark at 15°C for the indicated durations. Total proteins were subsequently extracted and used for the measurement of L-cysteine desulfhydrase (L-CDes, white bars), D-cysteine desulfhydrase (D-CDes, gray bars), and β-cyanoalanine synthase (β-CAS, dark bars) activities. Values are mean ± *S.E*. of three independent experiments. Asterisks represent statistical differences relative to the activity in dry seed protein extracts (Student's test; *P < 0.05; **P < 0.01); **(C)** Evolution of the activities of H_2_S-generating enzymes in Arabidopsis seeds imbibed on water or 1 mM NaHS. Seeds were imbibed on distilled water or 1 mM NaHS in the dark at 15°C for 24 h. Total proteins were subsequently extracted and used for the measurement of L-cysteine desulfhydrase (L-CDes), D-cysteine desulfhydrase (D-CDes), and β-cyanoalanine synthase (β-CAS) activities. Activities were compared with those of dry seeds. Values are mean ± *S.E.* of three independent experiments.

To get further information on the possible involvement of H_2_S in regulating germinative capacity, we assessed whether seeds processing to germination endogenously produced H_2_S during imbibition. As shown on Figure 2A, a slight increase (~40% compared to dry seeds) of H_2_S content was observed after 6 h imbibition and was maintained at 24 h. To evaluate the origin(s) of the H_2_S produced during seed imbibition, the activity of three enzymes reported to generate H_2_S in plants, i.e., L-cysteine desulfhydrase (L-CDes; E.C. 4.4.1.28), D-cysteine desulfhydrase (D-CDes; E.C. 4.4.1.15), and β-cyanoalanine synthase (β-CAS; E.C. 4.4.1.9), was compared in dry and imbibed seeds (Figure 2B). The three activities could be detected in protein extracts from dry seeds. All three activities were stimulated in imbibed seeds and reached a maximum after 24 h At this time point, activity was 3.7, 5.25, and 1.7 fold higher than in dry seeds, for L-CDes, D-CDes, and β-CAS, respectively. Nevertheless, the level of β-CAS activity remained ~10 fold lower than that of CDes activities suggesting that L- and D-CDes were likely the major sources of H_2_S during seed germination. Based on these observations, we investigated the impact of seed treatments with NaHS on L-CDes, D-CDes, and β-CAS activities. As shown on Figure 2C, the activities of the three enzymes were similar for seeds imbibed 24 h in the absence or presence of NaHS suggesting that the delay of seed germination triggered by high NaHS concentrations is not achieved via the modification of endogenous H_2_S metabolism.

### Impairment of H_2_S accumulation during imbibition delays seed germination

**Figure 3:**
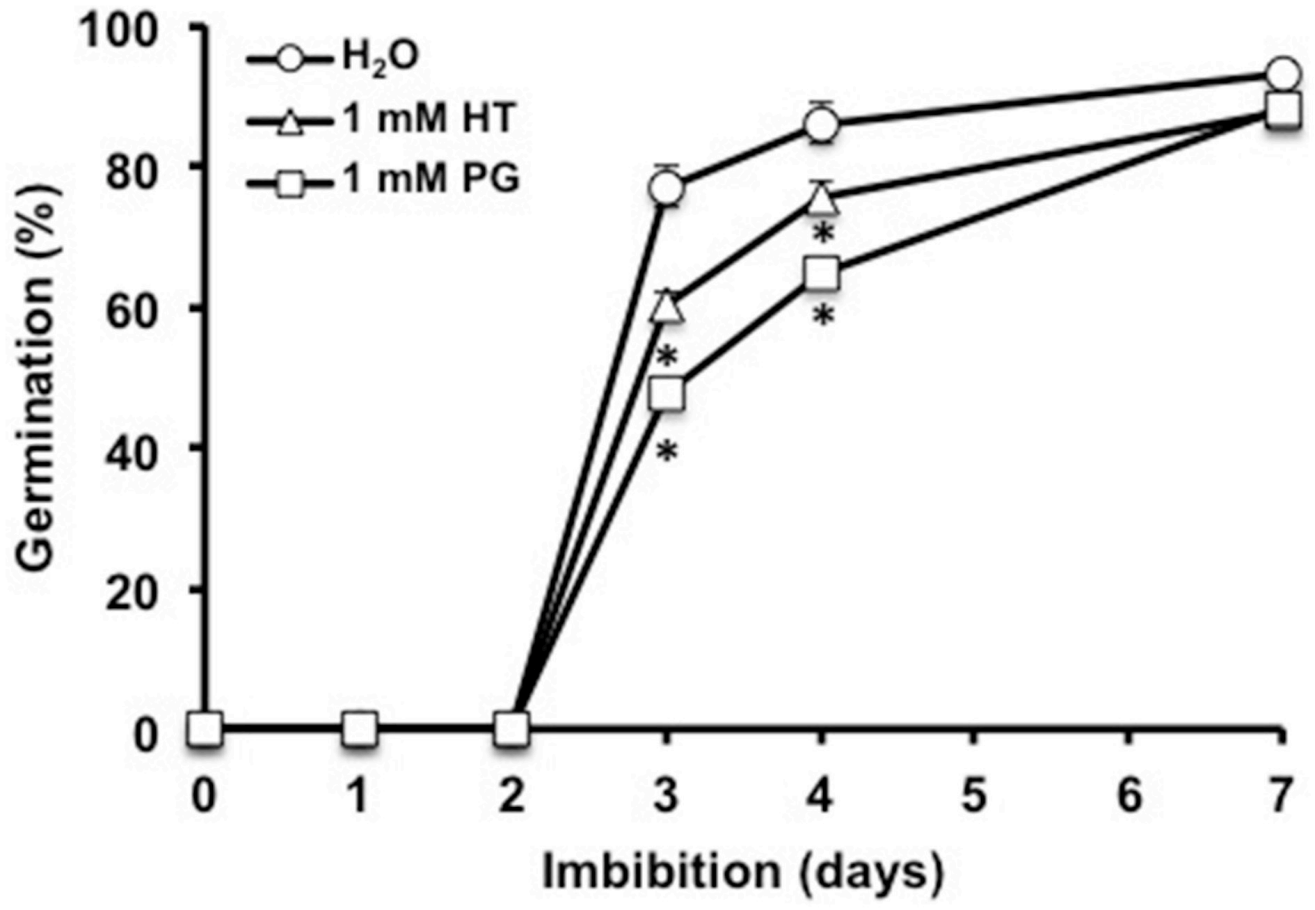
**Effect of inhibitors of H_**2**_S synthesis on Arabidopsis seed germination**. Seeds (50 per condition) were imbibed on paper filters soaked with distilled water (control, circles), 1 mM hypotaurine (HT, triangles) or 1 mM propargylglycine (PG, squares). Germination was recorded after incubation at 15°C in the dark for the indicated durations. Values are the mean ± *S.E*. of six experiments. Asterisks represent statistical differences relative to control at the same time point (Student's test; *P < 0.05).

To assess whether endogenous H_2_S might participate in regulating germination, seeds were germinated in the presence of hypotaurine (HT, a H_2_S scavenger) or DL-propargylglycine (PG, an inhibitor of CDes). After 3 days, germination was reduced by 25 and 40% by HT and PG, respectively (Figure 3). Nevertheless, HT and PG did not block, but only delayed germination, as comparable final rates of germination were reached after 7 days. These data indicate that impairing endogenous H_2_S formation impacts seed germination capacity.

### D-Cdes mutant seeds do not accumulate H_2_S during imbibition but exhibit unmodified germination

**Figure 4:**
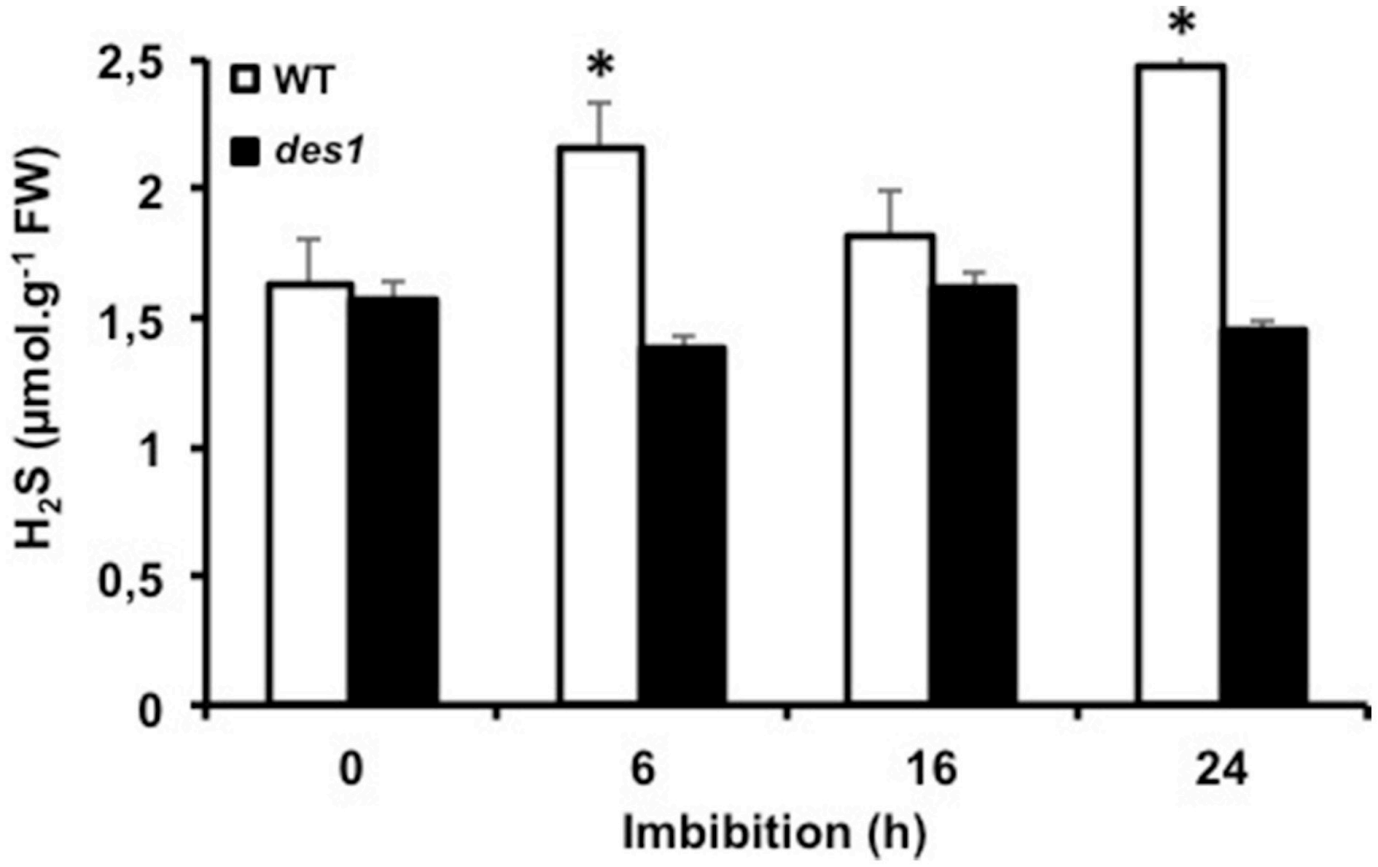
**Evolution of the endogenous H_**2**_S content of wild-type and *des1* seeds during imbibition**. Wild-type (white bars) and *des1* (dark bars) seeds were imbibed on distilled water in the dark at 15°C. Values are mean ± *S.E*. of three independent experiments. Asterisks represent statistical differences relative to H_2_S content in dry seeds (Student's test; *P < 0.05).

The impairment of germination by PG treatment together with the strong increase of CDes activities during seed imbibition suggested the involvement of CDes in H_2_S generation in imbibed seeds. A mutant line for *DES1* gene (*des1-1*) deficient for the sole cytosolic L-CDes characterized to date (Alvarez et al., 2010) was therefore analyzed for H_2_S production in imbibed seeds. The H_2_S content of freshly harvested *des1* seeds was compared with that of WT seeds at the dry state or after different durations of imbibition at 15°C (Figure 4). Comparable H_2_S contents were measured in WT and *des1* dry seeds. In contrast, whereas H_2_S content raised in WT seeds after imbibition, it remained unmodified over 24 h of imbibition in *des1* seeds, therefore implicating DES1 as a major source for H_2_S in germinating Arabidopsis seeds.

**Figure 5:**
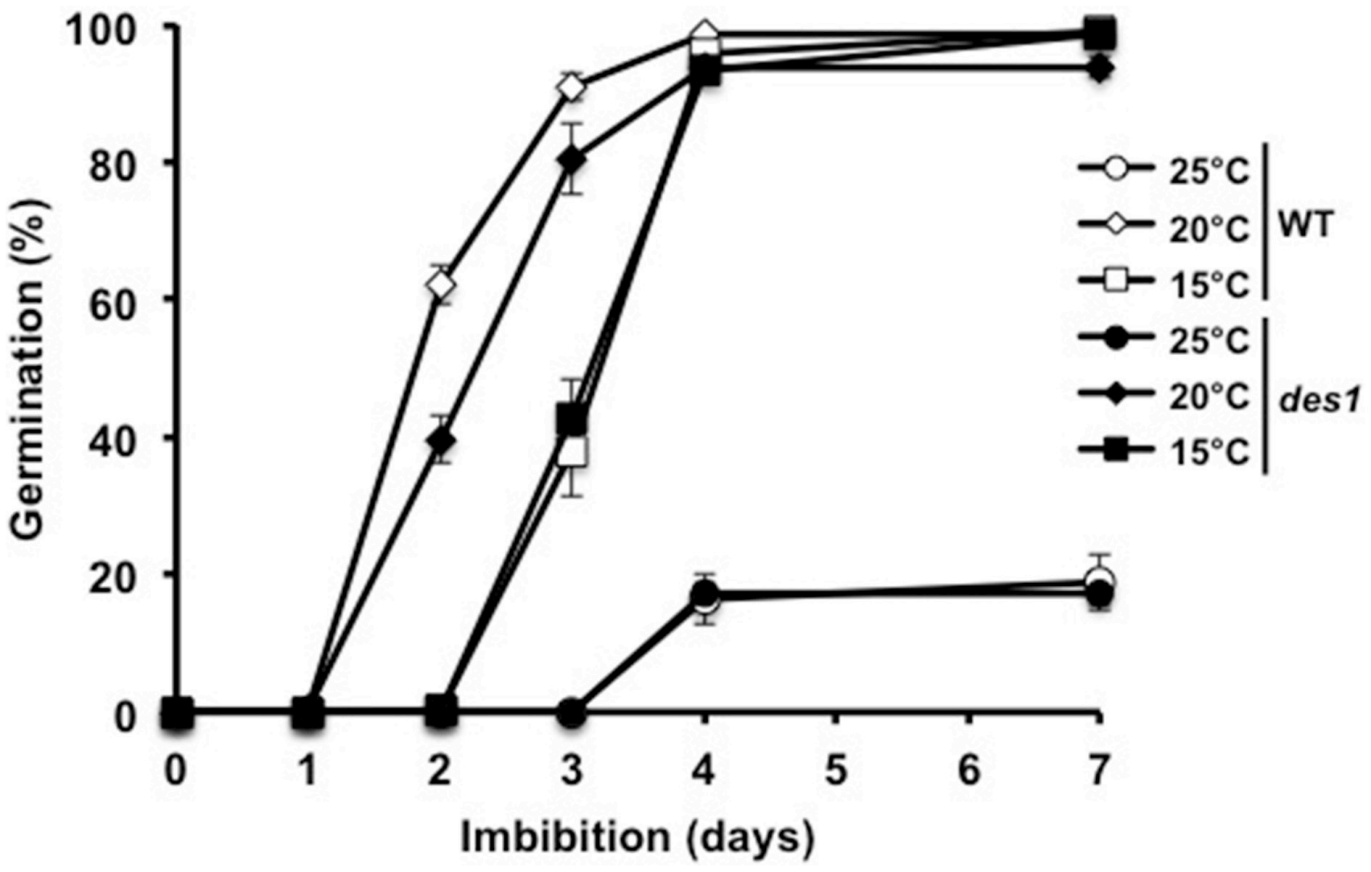
**Effect of temperature on wild-type and ***des1*** seed germination**. Wild-type (open symbols) and *des1* (closed symbols) seeds were imbibed on distilled water and germinated in the dark at 15°C (squares), 20°C (diamonds), or 25°C (circles). Values are mean ± *S.E*. of six experiments.

**Figure 6:**
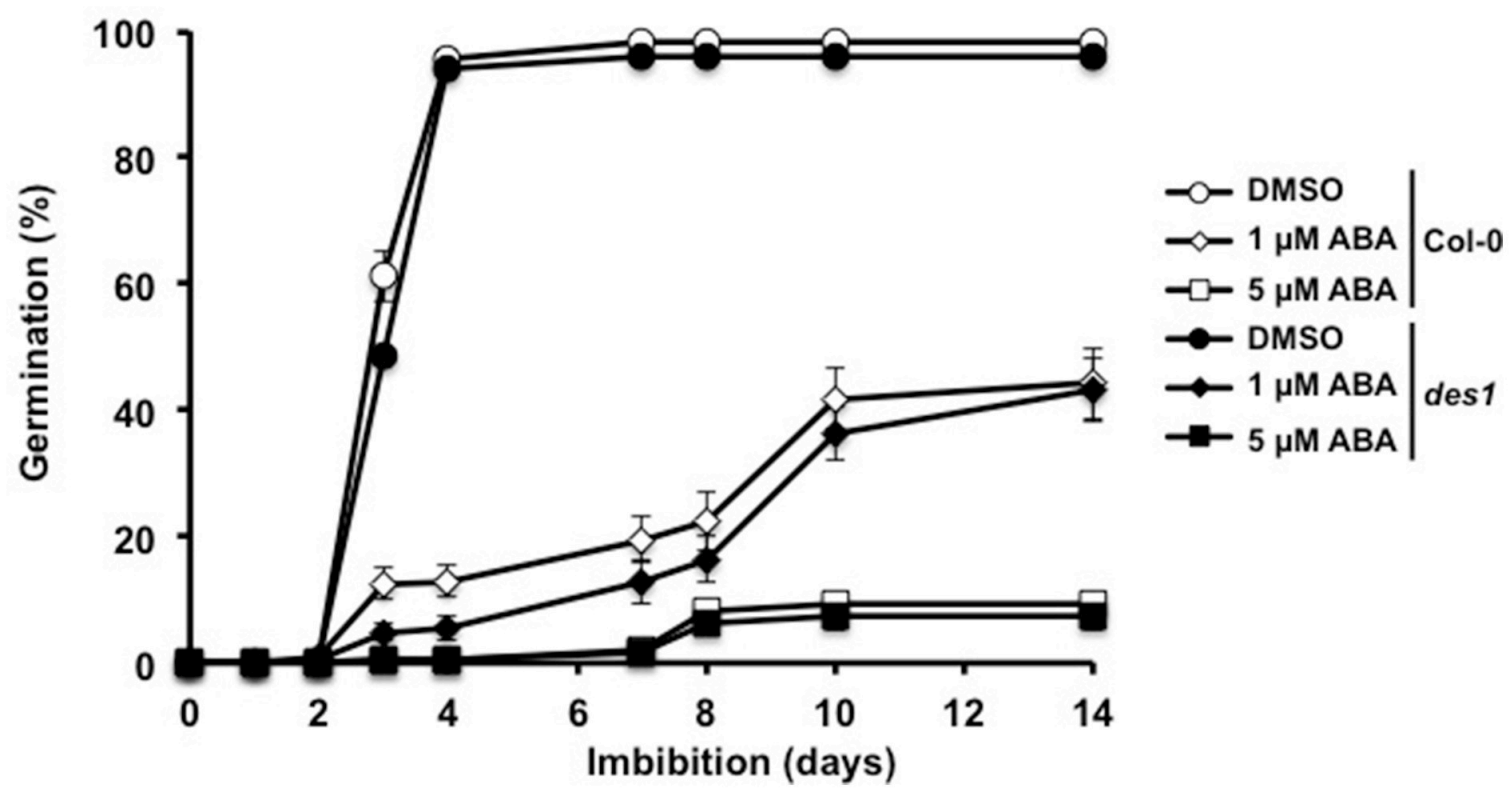
**Effect of ABA on wild-type and ***des1*** seed germination**. Wild-type (open symbols) and *des1* (closed symbols) seeds were imbibed on distilled water containing 1% DMSO (circles), 1 μM ABA (diamonds) or 5 μM ABA (squares). Seeds were subsequently incubated at 15°C in the dark. Values are mean ± *S.E*. of six experiments.

We further compared WT and *des1* mutant seeds under conditions known to influence germinative capacity. We first monitored the effect of temperature on the germination of both lines (Figure 5). When imbibed at 25°C, freshly harvested WT and *des1* seeds presented a low (~20%) percentage of germination, indicating that both seed lines were essentially dormant. At 15°C, the same lines fully germinated after 4 days. Imbibition at 20°C also led to full germination, with no significant difference between WT and *des1* seeds. These data therefore indicate that the mutation of *DES1* gene does not modify seed behavior when germinated at different temperatures nor than the dormancy of freshly harvested seeds. In addition, *des1* seeds presented a comparable sensitivity to NaHS treatments (Figure S1), which further suggests that the delay of germination triggered by NaHS is independent of endogenous H_2_S metabolism. As H_2_S has recently been evidenced as an intermediate of ABA signaling in leaf stomata, we compared the sensitivity of WT and *des1* seed germination to ABA. To achieve full and homogeneous germination of the two lines in the absence of ABA, the assays were run at 15°C. As shown on Figure 6, the germination of WT and *des1* seeds was sensitive to ABA. Indeed, the germination rate of WT and *des1* seeds was reduced by 60 and 90% in the presence of 1 and 5 μM ABA respectively. Therefore, the mutation of *DES1* gene does not modify the sensitivity of seed germination to ABA.

## Discussion

Recent years have provided an array of studies evidencing the biological effects of H_2_S in plants (Lisjak et al., 2013; Jin and Pei, 2015). Among them several reports suggested that H_2_S stimulated seed germination (Zhang et al., 2008, 2010a,b; Li et al., 2012; Dooley et al., 2013). Nevertheless, the observations essentially evidenced the capacity of H_2_S to alleviate the negative effects of stresses on germination, but were poorly informative on the effect of H_2_S on germination *per se*. Using Arabidopsis seeds as a model, we investigated the involvement of H_2_S in regulating germination under standard conditions. As reported in several physiological contexts including plant response to auxin (Fang et al., 2014), salt, and osmotic stress (Christou et al., 2013) or hypoxia (Cheng et al., 2013), we observed a significant increase of H_2_S content in seeds during imbibition. This increase occurred after 6 h and was maintained over 24 h. Increased production of H_2_S had been observed previously in seeds submitted to Al and osmotic stress (Zhang et al., 2010a,b). Our data indicate that it also occurs under standard germination conditions in the absence of stressing factors. It therefore suggested that endogenously evoked H_2_S might function as a signal during germination.

Different sources for H_2_S production have been reported in plants (Romero et al., 2014). In relation with seed physiology, Xie et al. (2014) correlated the variations of H_2_S content in wheat aleurone layers with the activity of L-cysteine desulfhydrase (L-CDes), the major source of H_2_S in plants (Alvarez et al., 2010). We could measure a L-CDes activity (27 ± 2 nmol H_2_S.min^−1^.mg prot^−1^) in dry Arabidopsis seeds in the range of those reported for plant tissues (10–150 nmol H_2_S.min^−1^.mg prot^−1^) (Riemenschneider et al., 2005a; Alvarez et al., 2010; Hou et al., 2013; Fang et al., 2014; Xie et al., 2014). L-CDes activity was strongly enhanced during imbibition, reaching a maximum after 24 h. It could therefore afford for the higher H_2_S contents found in imbibed seeds. We also observed a concomitant increase of the activity of D-cysteine desulfhydrase (D-CDes) and β-cyanoalanine synthase (β-CAS), two other enzymes generating H_2_S in plants (Riemenschneider et al., 2005a; García et al., 2010). Because of the toxicity of H_2_S for cytochrome c oxidase, it is unlikely that H_2_S generated by the mitochondrial β-CAS could accumulate to significant levels *in planta*, and the higher β-CAS activity is therefore likely related to cyanide detoxification (Álvarez et al., 2012b). D-CDes that exhibits a strong activity in imbibed seeds could generate part of the H_2_S measured in seeds, assuming that D-cysteine levels are sufficient (Riemenschneider et al., 2005b). Nevertheless, our study indicates that L-CDes is critical for H_2_S production in imbibed seeds. Indeed, knock-out mutant seeds for the *DES1* gene that encodes the cytosolic L-CDes (Alvarez et al., 2010) presented similar H_2_S contents in dry and imbibed seeds. The stimulation of DES1 activity is therefore the main route for the enhanced H_2_S production observed in imbibed seeds. On the other hand, the basal H_2_S level found in dry seeds and detected in imbibed *des1* mutant seeds might be related to β-CAS and/or D-CDes activities. It might also be due to unidentified L-CDes enzymes as suggested by the significant L-CDes activity retained by the *des1* mutant (Alvarez et al., 2010).

To decipher the requirement and possible function of H_2_S during seed germination, we used chemicals either artificially releasing H_2_S (NaHS) or inhibiting endogenous H_2_S formation (hypotaurine and propargylglycine). No effect of NaHS was observed at low concentrations (≤100 μM), whereas germination was significantly delayed at higher concentrations. This effect is likely due to a massive and rapid release of H_2_S from NaHS toxic for the plant material. Indeed no such retardation was observed with GYY4137, that releases lower H_2_S doses (Lisjak et al., 2010), and GYY4137 did not affect Arabidopsis seed germination at any concentrations tested up to 1 mM (data not shown). Our data further illustrate contrasted effects of H_2_S on seed germination observed under standard conditions. Indeed, no effect was found on wheat seed germination in the presence of up to 1.5 mM NaHS (Zhang et al., 2010a). On the other hand, Dooley et al. (2013) reported a stimulation of seed germination by H_2_S in diverse plant species including corn, pea, bean, and wheat. Different sensitivities toward stimulators/inhibitors of germination are frequently observed between species and might afford for the different response to H_2_S treatment in different species (Finch-Savage and Leubner-Metzger, 2006). In addition the response might depend on the conditions used for treatment and germination (light status, temperature…) that are varying between studies.

To get further insights in the function of endogenously evoked H_2_S, we used hypotaurine and propargylglycine to modulate H_2_S content in seeds. Hypotaurine (HT) is a potent H_2_S scavenger (Ortega et al., 2008) and has been used in plants to lower intracellular H_2_S content (Li et al., 2014). Propargylglycine (PG) inhibits CDes activities (Steegborn et al., 1999) and blocks H_2_S production in plants (Li et al., 2014). When applied during seed imbibition both compounds strongly delayed germination. As their modes of action are different this effect is certainly reached through the blocking of H_2_S formation. It therefore supports that endogenous H_2_S formation is required for optimal germination. Noteworthy the effect of HT and PG was similar to that of high NaHS treatments, which questioned on the possible impact of NaHS application on seed H_2_S metabolism. Although the mechanisms by which high NaHS concentrations delay germination are unknown, our data indicate that they do not rely on modifications of endogenous H_2_S metabolism. Indeed seeds imbibed in the presence or absence of NaHS exhibit similar L-CDes, D-CDes, and β-CAS activities. Moreover, the germination of *des1* seeds was as sensitive as WT seeds to NaHS. These observations are consistent with previous data by Riemenschneider et al. (2005a) indicating that L-CDes and D-CDes activities were not affected in H_2_S-fumigated Arabidopsis plants. On the other hand optimal germination might require a particular level of endogenous H_2_S that would be unbalanced by exogenous NaHS, HT, and PG treatments. This phenomenon has been proposed for H_2_O_2_ for which too low or too high intracellular levels both lead to germination impairment (Bailly et al., 2008). In any case, only a delay of germination, and not a full inhibition, has been observed for the different treatments. As long-term effects of HT and PG have been reported with lower concentrations than the one used in our study (Li et al., 2014), it is unlikely that HT and PG loose their efficiency over time. It more likely reflects that endogenously evoked H_2_S, although fastening germination, is not a requisite. This hypothesis is strengthened by the fact that *des1* mutant seeds germinate at similar rates compared to WT seeds. Experiments run at 15°C indicate that DES1 activity, and more generally endogenously evoked H_2_S, is dispensable for germination. Similarly, because both *des1* and WT seeds hardly germinate at 25°C, DES1-dependent H_2_S production is not required for seed dormancy. In good accordance with this, *des1* and WT seeds exhibited comparable sensitivity toward ABA, which is the major regulator of seed dormancy. Contrarily to stomata that exhibit an altered response to ABA in *des1* mutant and in which DES1-dependent H_2_S formation is an intermediate of ABA signaling (Scuffi et al., 2014), our data indicate that ABA signaling in seeds is independent of DES1. More globally, as no altered development has been observed for *des1* seedlings (Alvarez et al., 2010; Jin et al., 2013), our data further highlight that DES1 is dispensable during the early stages of plant development under optimal growth conditions.

Taken together our data highlight that although H_2_S is produced at the early stages of seed imbibition essentially via the activity of L-CDes, its importance, if any, for germination under standard conditions is limited in Arabidopsis. This contrasts with other reactive species such as hydrogen peroxide or nitric oxide that are now considered as major regulators of seed germination and seed dormancy release (Arc et al., 2013; Diaz-Vivancos et al., 2013). As treatments with H_2_S donors efficiently alleviate inhibition of germination by abiotic stresses, future works should be focused on deciphering whether endogenously evoked H_2_S participates in abiotic stress tolerance during seed germination and could therefore constitute a trait for variety selection and improvement.

## Author contributions

EB, JP, and CB designed the research. EB, AP, NI, and FC carried out the experiments and analyzed the data. EB, JP, and CB contributed to writing the manuscript. EB and CB supervised the project.

## Funding

This work was supported by the Centre National de la Recherche Scientifique and the Université Pierre et Marie Curie- Paris 6.

### Conflict of interest statement

The authors declare that the research was conducted in the absence of any commercial or financial relationships that could be construed as a potential conflict of interest.

## Supplementary material

Figure S1 **Effect of NaHS treatments on wild-type and ***des1*** seed germination**. WT and *des1* seeds (50 per condition) were imbibed on paper filters soaked with distilled water containing 1 mM Na_2_SO_4_ (0) or increasing concentrations of NaHS. Germination was recorded after incubation at 15°C in the dark for 4 days. For all the conditions, 98–100% germination was achieved after 7 days. Values are the mean ± *S.E*. of three experiments.
