# Supplementary figures and images for: The Significance of Hydrogen Sulfide for Arabidopsis Seed Germination
Source: Front Plant Sci. 2016 Jun 27;7:930. doi: 10.3389/fpls.2016.00930 (PMC4921499; doi:10.3389/fpls.2016.00930)

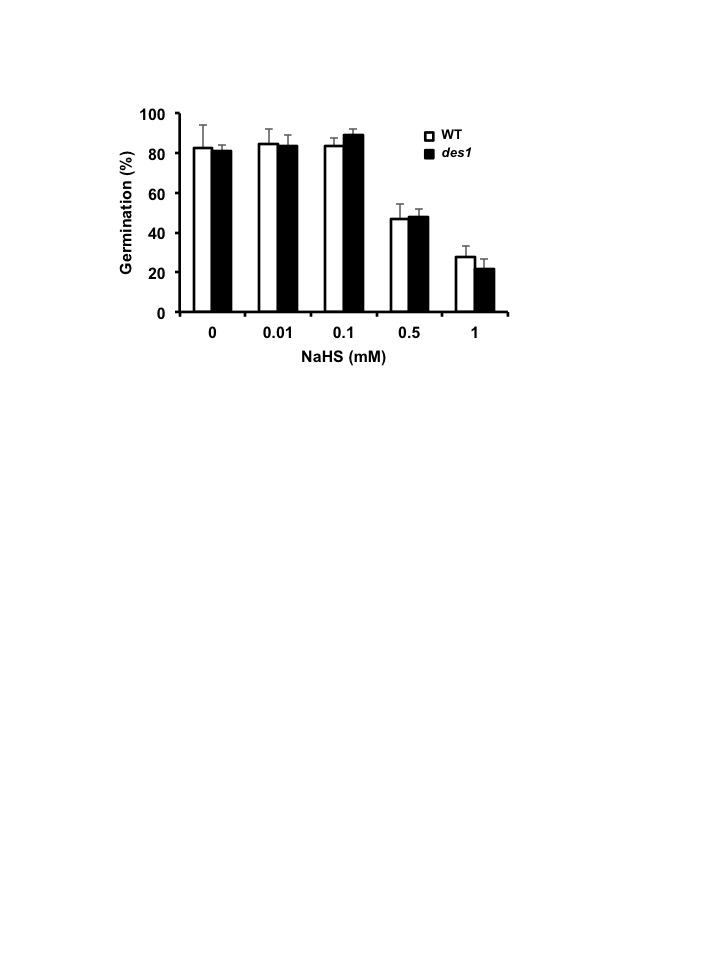

Supplement: Figure S1 — Effect of NaHS treatments on wild-type and des1 seed germination. WT and des1 seeds (50 per condition) were imbibed on paper filters soaked with distilled water containing 1 mM Na2SO4 (0) or increasing concentrations of NaHS. Germination was recorded after incubation at 15°C in the dark for 4 days. For all the conditions, 98–100% germination was achieved after 7 days. Values are the mean ± S.E. of three experiments. [file Image1.JPEG]
